# Analysis of the grape *MYB R2R3* subfamily reveals expanded wine quality-related clades and conserved gene structure organization across *Vitis* and *Arabidopsis* genomes

**DOI:** 10.1186/1471-2229-8-83

**Published:** 2008-07-22

**Authors:** José Tomás Matus, Felipe Aquea, Patricio Arce-Johnson

**Affiliations:** 1 Departamento de Fruticultura y Enología, Pontificia Universidad Católica de Chile, Av. Vicuña Mackenna 4560, Santiago, Chile; 2 Departamento de Genética Molecular y Microbiología, Pontificia Universidad Católica de Chile, Alameda 340, PO Box 114-D, Santiago, Chile

## Abstract

**Background:**

The MYB superfamily constitutes the most abundant group of transcription factors described in plants. Members control processes such as epidermal cell differentiation, stomatal aperture, flavonoid synthesis, cold and drought tolerance and pathogen resistance. No genome-wide characterization of this family has been conducted in a woody species such as grapevine. In addition, previous analysis of the recently released grape genome sequence suggested expansion events of several gene families involved in wine quality.

**Results:**

We describe and classify 108 members of the grape *R2R3 MYB *gene subfamily in terms of their genomic gene structures and similarity to their putative *Arabidopsis thaliana *orthologues. Seven gene models were derived and analyzed in terms of gene expression and their DNA binding domain structures. Despite low overall sequence homology in the C-terminus of all proteins, even in those with similar functions across *Arabidopsis *and *Vitis*, highly conserved motif sequences and exon lengths were found. The grape epidermal cell fate clade is expanded when compared with the *Arabidopsis *and rice MYB subfamilies. Two anthocyanin *MYBA *related clusters were identified in chromosomes 2 and 14, one of which includes the previously described grape colour locus. Tannin related loci were also detected with eight candidate homologues in chromosomes 4, 9 and 11.

**Conclusion:**

This genome wide transcription factor analysis in *Vitis *suggests that clade-specific grape *R2R3 MYB *genes are expanded while other MYB genes could be well conserved compared to *Arabidopsis*. *MYB *gene abundance, homology and orientation within particular loci also suggests that expanded MYB clades conferring quality attributes of grapes and wines, such as colour and astringency, could possess redundant, overlapping and cooperative functions.

## Background

The MYB superfamily exemplifies how both conserved and divergent domains are present within a transcription factor family. *MYB *genes are exclusive to eukaryotes [1,2] and although in animals their function is restricted to the control of the cell division and differentiation [3], they have diverse functions in plants [4].

*MYB *genes in plants are modulated by diverse hormones [5] and participate in key processes related to epidermal cell destiny [6] (which includes pigmentation [7] and formation of trichomes [8]), seed development [9], response to drought [10] and cold [11,12], pathogen-disease resistance [13-15] stomatal movements [16,17], phytochrome A-dependent light-sensing responses [18] and sucrose related responses [19], among many other functions.

Different MYB families have evolved after duplications of their DNA binding domains [4]. MYB DNA binding domains are 100–160 residues in length, depending on the number of imperfect repeats (named R) in the N-terminal region. From the different classes identified, the R2R3 subfamily is the most abundant in plants [20-22]. Each repeat adopts a helix-helix-turn-helix structure which interacts with regulatory elements in the promoter, while the C-terminal region is responsible for establishing protein-protein interactions with other components of the eukaryotic transcriptional machinery. Based on their well conserved DNA-binding domains, *R2R3 MYB *genes have been annotated in both monocotyledonous and dicotyledonous genomes. In *Arabidopsis thaliana*, there are an estimated 126 R2R3 subfamily members [5], although only some of them have been functionally characterised.

Despite MYB genes have also been identified in woody species such as poplar, grape and apple, no genome-wide characterization of this family has been conducted in such species. In grapes, the *MYB *genes characterised to date are all involved in the control of flavonoid synthesis (*MYBA *[23], *MYB5a *(*MYBCS-1*) [24], *MYB5b *[25], and *MYBPA1 *[26]). Since quality parameters in grapes and wines such as colour, bitterness and astringency are determined by flavonoid compounds accumulating in fruit tissues, these studies have focussed on anthocyanin and proanthocyanidin synthesis and their regulation via MYB factors. In species such as *Arabidopsis*, petunia and maize, it has been determined that MYB proteins can act together with bHLH and WDR proteins in a transcriptional complex capable of regulating flavonoid synthesis and other processes concerning epidermal cell identity [27,28]. The affinity of MYB proteins for the DNA binding sites in each promoter can be influenced by the interaction with these factors.

The annotated genome sequence of *Vitis vinifera *has recently become available [29] after an Italian-French effort to obtain a highly homozygous Pinot noir (PN40024) genome [30]. In addition, an Italian consortium sequenced a highly heterozygous Pinot noir genotype (ENTAV115) [31,32]. These two initiatives provided a genomic platform for studying this fruit crop. To date, its sequence has been assigned to 19 chromosomes [30] or linkage groups [32], the same number as in Poplar (*Populus trichocarpa*) [33]. While the PN40024 genotype is predicted to contain 30,434 genes in 487 Mb (46% of the genome; [30]), the ENTAV115 clone is predicted to have 29,585 genes within 531 Mb [32]. Although some supercontigs are still being correctly assembled and oriented in each chromosome, the sequence shows that 41.4% of the genome is composed of transposable elements mostly located in introns, explaining its high variability and heterozygocity [30]. Paralogous regions in the grape genome arose by gene duplication and are present in clusters, forcing gene expansion and diversification. The best example of this event is the terpene synthase gene family, with 30–40 members in *Arabidopsis *and almost 90 members in grape [30]. To date, information regarding expansion events of the *MYB *gene superfamily in *Vitis *is absent.

In this work, we describe the putative *R2R3 MYB *gene subfamily by means of *in silico *analysis of the grape genome sequence, in order to predict protein domain architectures, and to assess the extent of conservation and divergence between grape and *Arabidopsis *gene families. Candidate genes were chosen for isolation and their expressions were tested in different grape organs to compare expression patterns of closely grouping co-orthologues. This extended analysis is the first to be performed on a dicotyledonous species other than *Arabidopsis*. Possible gene retention, loss and expansion processes of *MYB *genes are discussed.

## Results and Discussion

### Identification and chromosomal distribution of grape R2R3 MYB genes

The genome sequence of the homozygous PN40024 genotype of *Vitis vinifera *cv. Pinot noir [29] was searched for *MYB *gene models. These are predicted from combining *ab initio *models together with *V. vinifera *complementary DNA sequences (from EST databases) and alignments of gene/protein models from other species [30]. A consensus R2R3 MYB DNA binding domain sequence (Additional file 1) was used in a BLATsearch, which maintains an index of the entire genome in memory and finds sequences of 20 amino acids or more with 80% or higher similarity in the genome. The MYB superfamily has been defined as the most abundant transcription factor family in plants [34], with at least 198 members in *Arabidopsis *and 183 in rice [5]. In grapevine, a total of 279 *MYB *genes were previously estimated [32], including members of the *R1R2R3*, *R2R3*, atypical *MYB *and *MYB-related *gene subfamilies. The *Arabidopsis *and rice R2R3 subfamilies are composed of 126 and 109 members, respectively [5], while in grape 108 candidate genes were identified in this study. These were further analysed in terms of their putative protein sequences, exon lengths and their positions and orientations in each chromosome (Additional file 2).

**Figure 1:**
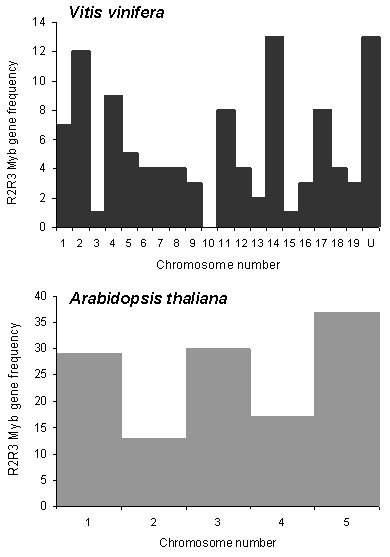
**Representation of *Vitis *and *Arabidopsis *genomes regarding R2R3 MYB gene frequency per chromosome**. A R2R3 consensus sequence was used in a Blat Search in the Grape Genome Browser [29] in order to identify gene models with R2R3 MYB like identities. *Arabidopsis *chromosome MYB abundances were obtained from TAIR.

This analysis revealed that grape *MYB *genes are distributed in almost all chromosomes, except for chromosome (chr) 10 (Figure 1). However, thirteen *MYB *gene models have not yet been assigned to a particular chromosome and it is possible that some of them are positioned in chr10. Chromosomes with more abundant *MYB *gene models are 1, 3 and 5 in *Arabidopsis *and 2, 4 and 14 in the *Vitis *genome. These were subsequently analysed for gene cluster expansion events.

### Exon/intron organisation of Vitis and Arabidopsis MYB families

**Figure 2:**
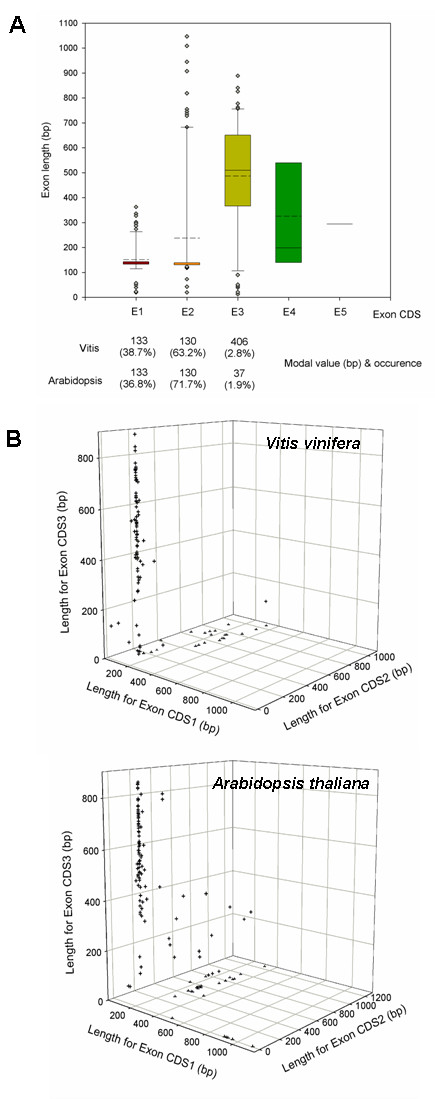
**Exon length distribution analysis of the grape *R2R3 MYB *gene models**. A) Exon length values were analysed using Boxplot. Each box represents the exon size range in which 50% of the values for a particular exon are grouped. The mean value is shown as a dotted line and the median as a continuous line. Only eight gene models possess four exons while two genes are predicted to have five. B) Distribution of grape and *Arabidopsis MYB *genes regarding their first, second and third exon lengths.

**Figure 3:**
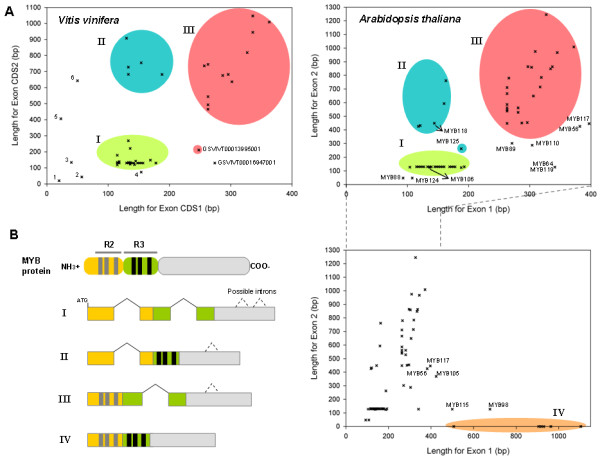
**Exon structure and protein domain relationships of *Arabidopsis *and grape R2R3 MYBs**. A) Scatter diagram of 108 and 126 *MYB *genes from *Vitis *and *Arabidopsis*, respectively. First and second exon lengths were projected on the first and second axis, respectively. B) Classification of each group from the scatter plot (I-IV) regarding the protein domain boundaries encoded by each exon. Grape spots not included in any group correspond to the incomplete R2R3 gene models 1: GSVIVT00032266001, 2: GSVIVT00030108001, 3: GSVIVT00038769001, 4: GSVIVT00008781001, 5: GSVIVT00016942001 and 6: GSVIVT00006679001. Some *Arabidopsis MYB *genes are not included in any group due to their complex exon/intron organisation structure (see Results and discussion section).

Exon structures are highly conserved when the grape and *Arabidopsis *R2R3 MYB subfamilies are compared (Figures 2 and 3). Exons 1 and 2 code for almost the entire R2R3 DNA binding domain, although this pattern differs in complex multiexonic genes (e.g. *AtMYB88 *and *124*). As shown in Figure 2A, exon 1 appears to be the most restricted in length, while exons 2 and 3 are more variable (<100 bp – >1000 bp). Presence of a fourth and fifth exon is exclusive to some particular genes. Despite this variability, the modal lengths of the first two exons are very similar (exon 1, 133 bp; exon 2, 130 bp) and highly conserved (exon 1, 38.7% occurrence; exon 2, 63.2% occurrence). These occurrence values are very similar in *Arabidopsis*, suggesting that MYB DNA binding domains could be partially conserved because exons coding for this domain have restricted lengths during the evolution of plant species. It is interesting to note that the exon lengths observed for the R2R3 MYB subfamily coincide with the average exon length predicted from models of other grape gene families (130 bp, [30]).

Although exon 3 is the most diverse in size, MYB subfamilies from both species are similarly distributed when the first three exon lengths are considered (Figure 2B). Exon 3 codes for the last region of the R3 repeat and for the C-region of the protein. Changes both in length and sequence of this exon could have generated functional divergence between MYB homologues within and between species, leading to different functional motifs and domains [35].

In order to gain further insights into exon conservation, the split positions of the R2R3 repeats within the DNA binding domain were classified in the grape and *Arabidopsis *gene models (Figure 3; note that this classification does not consider the final number of exons). It is possible to distinguish four groups in *Arabidopsis*, the first three of which are also present in grape (Figure 3A). All groups differ in the organisation of exons determining the R2R3 domain. In general, the R2R3 domain consists of 106 amino acids. The N-terminal region before this domain may vary within each group. Groups I and II have their R2 repeat split between exons 1 and 2, but in Group I, the R3 repeat is divided between exons 2 and 3 (Figure 3B; note that the last exon in Groups I, II and III may have additional introns depending on the gene). In Group III, the first exon codes the complete R2 repeat but R3 is split between in exons 1 and 2. Group IV consists only of 1 exon coding for the complete MYB protein: members of this group have not been identified to date in grapes. *AtMYB88 *and *AtMYB124 *are not included in any group of the *Arabidopsis *plot because of their more complex exon/intron organization (10–11 exons, respectively).

Seven *Arabidopsis MYB *genes and the grape gene model GSVIVT16947001 could not be positioned in these groups. Together with *MYB118 *and *106*, all share a longer N-terminal region before the start of the R2 repeat (between 52 and 213 residues). Regarding their exon boundaries, the R2 and R3 repeats are organised differently and can not be classified by these means. The other *Arabidopsis MYBs *and their grape gene homologues are maintained in the same respective groups. Despite the occurrence of different duplication and diversification events in these two species, *MYB *exons are highly conserved. Length and sequence restrictions in the first two exons could help maintain functionality and achieve sequence specificity of the R2R3 domain, while variations in the exons coding for the C-terminal regions could facilitate the gaining of new or cooperative functions when new domains are included.

#### Updated functional clustering of the Arabidopsis R2R3 MYB Subfamily

**Figure 4:**
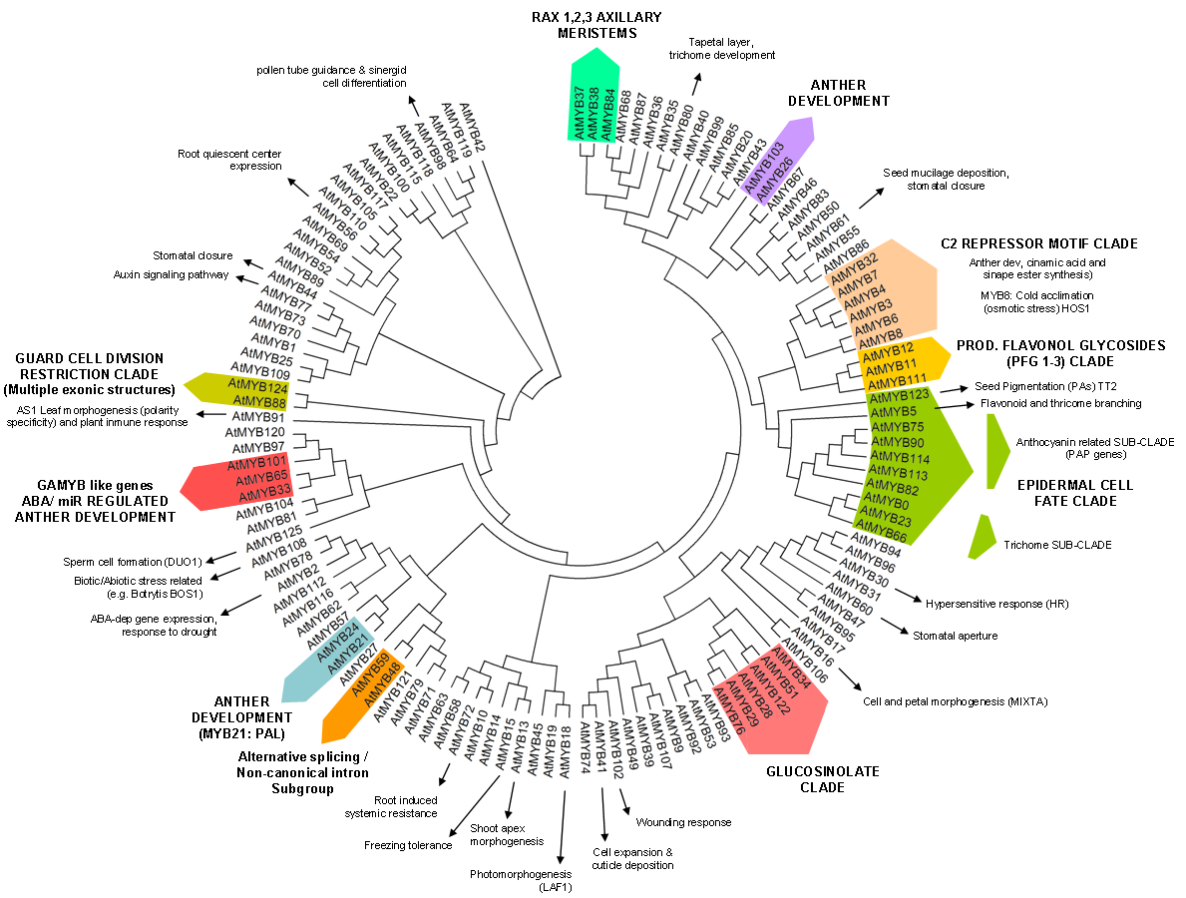
**Integrated evolutionary relationships of the 126 *Arabidopsis *R2R3 MYB proteins**. Consensus circular rooted tree was inferred by Neighbour-Joining method and 1000 bootstraps using Mega4 software. Each functional clade is highlighted. References for *MYB *gene functions are also shown in Additional file 3.

Taking the complete *Arabidopsis *R2R3 MYB subfamily (126 members, Additional file 3), a phylogenetic tree was constructed to update new functional clades which could also be present in the grape subfamily (Figure 4). Most genes sharing similar functions were clustered in the same phylogenetic clades and subclades (functional clades), suggesting that most closely-related MYBs in the same species could recognise similar target genes and possess redundant, overlapping, and/or cooperative functions.

The resulting tree is validated as it shows the same subgroups (1–17) observed in phylogenetic trees constructed by Kranz et al [36] and Yanhui et al [5]. New functions have been recently identified, including MYBs involved in glucosinolate biosynthesis [37-41] and hormone induced responses. These new functions are also clustered within this phylogenetic analysis (Figure 4), although a particularly polyphyletic feature within this Subfamily is observed: flower development. Flower organs were conceived as an adaptive process late in plant evolution. Several mechanisms involving cell cycle regulation, secondary metabolite accumulation and hormonal control have been related to reproductive male organ development. *MYB *genes regulating these events have been recently identified (*AtMYB103 *[42,43], *AtMYB26 *[44], *AtMYB24 *[45], *AtMYB21 *[46], *AtMYB32 *[47] and *AtMYB101*, *AtMYB65 *and *AtMYB33 *[48,49]). Although they cluster in different clades within the MYB subfamily and their target genes seem to be different, all ultimately regulate anther development.

The R2R3 domain provides both the MYB nuclear localisation signals [50] and regulatory specificity [51]. C-terminal regions often possess protein-protein interaction or DNA-repression motifs. Such is the case of the C2 motif repressor clade. Members of this group possess the C1 and C2 motifs, known to participate in bHLH interactions [52] and promoter repression, respectively (e.g. CH4 promoter for AtMYB4; [52] and RD29A promoter for AtMYB8; [12]).

Among flavonoids, flavonols are known to have functions such as filtering UV radiation and auxin retention and transport (reviewed in [53]). They also have pharmaceutical properties when consumed by animals. Recently, their synthesis has been shown to be regulated by three closely related MYBs (AtMYB11, 12 and 111 [53]). These MYBs are found together in the 'production of flavonol glycosides' clade (Figure 4) and act at different times and tissues throughout plant development [53]. This new clade appears to share an ancestral MYB gene both with the 'C2-repressor motif' clade and the 'epidermal cell fate' clade (which includes the anthocyanin and trichome related subclades). These clades regulate different processes but share up to some extent the control of phenolic compound synthesis.

### Phylogenetic analysis of the grape R2R3 MYB Subfamily

The same alignment protein weight matrices and phylogeny algorithms used for the construction of the *Arabidopsis *tree were employed for the integrated analysis of the *Arabidopsis*, rice and *Vitis *MYB subfamilies (Additional files 4 and 5). Grape protein models with a complete R2R3 domain (91 sequences), seven characterised grape proteins and 126 *Arabidopsis *homologues were included in the first phylogeny. The remaining grape models generally possessed an incomplete R2 repeat, so were not included in this analysis.

Sequence alignment and phylogenetic tree analysis using both the single R2R3 DNA-binding domain and the full predicted protein sequences were produced (Additional file 4A and 4B, respectively). Outside the DNA binding domain, MYB proteins had the greatest sequence divergence. Although a full sequence alignment could be highly sensitive to the dissimilarities in the C-terminal regions outside the conserved motifs, both trees are extremely similar regarding topology and bootstrap values. Possible orthologues between both species appear outside and inside the functional clades previously described for *Arabidopsis *(Additional files 4 and 5).

In some cases, it was easy to identify two putative orthologues in *Arabidopsis *and *Vitis*, since they were grouped in pairs within a clade. In other situations, grape and *Arabidopsis *homologues were clustered by species within a clade (e.g. proteins from the C2-repressor motif clade, with the exception of MYB4). Their functional characterisation will help prove which genes are orthologues within each clade.

None of the grape gene models were grouped within the *Arabidopsis *'glucosinolate' clade (Additional file 4). Several lines of evidence suggests that this clade resulted from a β-type duplication [5] which evolved independently in the order *Brassicales *as an adaptive response to herbivory [54,55], explaining its absence in rice and grape.

For several transcription factor families, linear correlations of abundance have been identified in *Arabidopsis *and *Vitis *[32]. In *Arabidopsis*, the MYB superfamily consists of 198 *R1R2R3*, *R2R3*, *MYB related *and *atypical MYB *genes [5], in contrast to the 279 estimated MYB superfamily members in *Vitis*. In addition, because of the anatomical and physiological differences between grapes and Arabidopsis, it can be suggested that some clades could be differentially expanded when comparing *Arabidopsis *and *Vitis *R2R3 subfamilies. Both inside and outside of some of these functional clades, grape gene expansions were observed as gene pairs or clusters (highlighted with asterisks in Additional file 4). Such is the case of the *Vitis*-specific MYB5a and MYB5b, the MYB4 gene pair within the C2-repressor motif clade, MYB101 ('GAMYB like/miRNA regulated' clade) and MYB 38/RAX1 ('axillary meristem' clade). Outside the functional clades, expanded MYBs are represented by dichotomous pairs of grape MYB15, 17, 35, 67 and 102 homologues, whose functions have yet to be determined. In the case of the 'production of flavonol glycosides' clade, one member (possibly MYB11) may be absent in grape. This could be a result of the loss of a clade member or a mis-annotation of the *Vitis *genome. It is possible that new *R2R3 MYB *genes could be identified in the future as annotations improve. For instance, *AtMYB88 *and *AtMYB124 *grape homologues, which belong to the 'guard cell division restriction' clade, have yet to be found, possibly because their complex exon/intron structure (10–11 exons) makes them difficult to identify during an annotation procedure.

### Expansion of grape MYB genes affecting wine quality parameters

After the sequencing of the grapevine genome, the expansion of large gene families involved in wine characteristics was observed [30]. Such was the case of the Stilbene Synthase Family, responsible for the synthesis of the phytoalexin resveratrol, and the Terpene Synthase Family, involved in aroma production. Other studies have also revealed that genes belonging to terpene and flavonoid metabolic pathways have suffered selective amplifications in the grape genome, in contrast to other plant genomes [32]. If this situation is evident for some biosynthetic genes of the grape phenylpropanoid pathway (*PAL*, *CHS*, *F3H*, *F3'5'H*, *FLS *and *LAR*), it is reasonable to expect that *MYB *genes controlling this pathway could also be expanded. Nevertheless, it is also possible to suggest that these genes were maintained in the grapevine genome by means of human domestication, and lost in other plant genomes. For example, many genes were lost in *Arabidopsis *after its two wide genome duplication (WGD) events [56].

Two different scenarios have been described regarding possible WGD episodes in grape: the first states there is no evidence for such event [30], while in the second, a recent WGD event might have occurred in close proximity to the *Vitis *speciation event [32]. In our analysis we observed that *MYB *genes from the 'epidermal cell fate' clade involved in the synthesis of molecules which confer quality to grapes and wines are largely expanded in the *Vitis *genome.

Grape and wine colour is defined by glycosylated anthocyanins. Any perturbation in their cytoplasmic synthesis, conjugation or vacuole transport thus directly affects berry colour. Several *MYB *genes have been characterised in *Arabidopsis*, maize, petunia, antirrhinum, and apple, which regulate some of the processes concerning anthocyanin production. It was discovered that white grapes arose from multiallelic mutations of the *VvMYBA1/A2 *genes [57-60], which controls the last biosynthetic step of anthocyanin synthesis, a glycosylation reaction mediated by the UDP-GLUCOSE FLAVONOID 3-O-GLUCOSYLTRANSFERASE (UFGT) enzyme [23].

**Figure 5:**
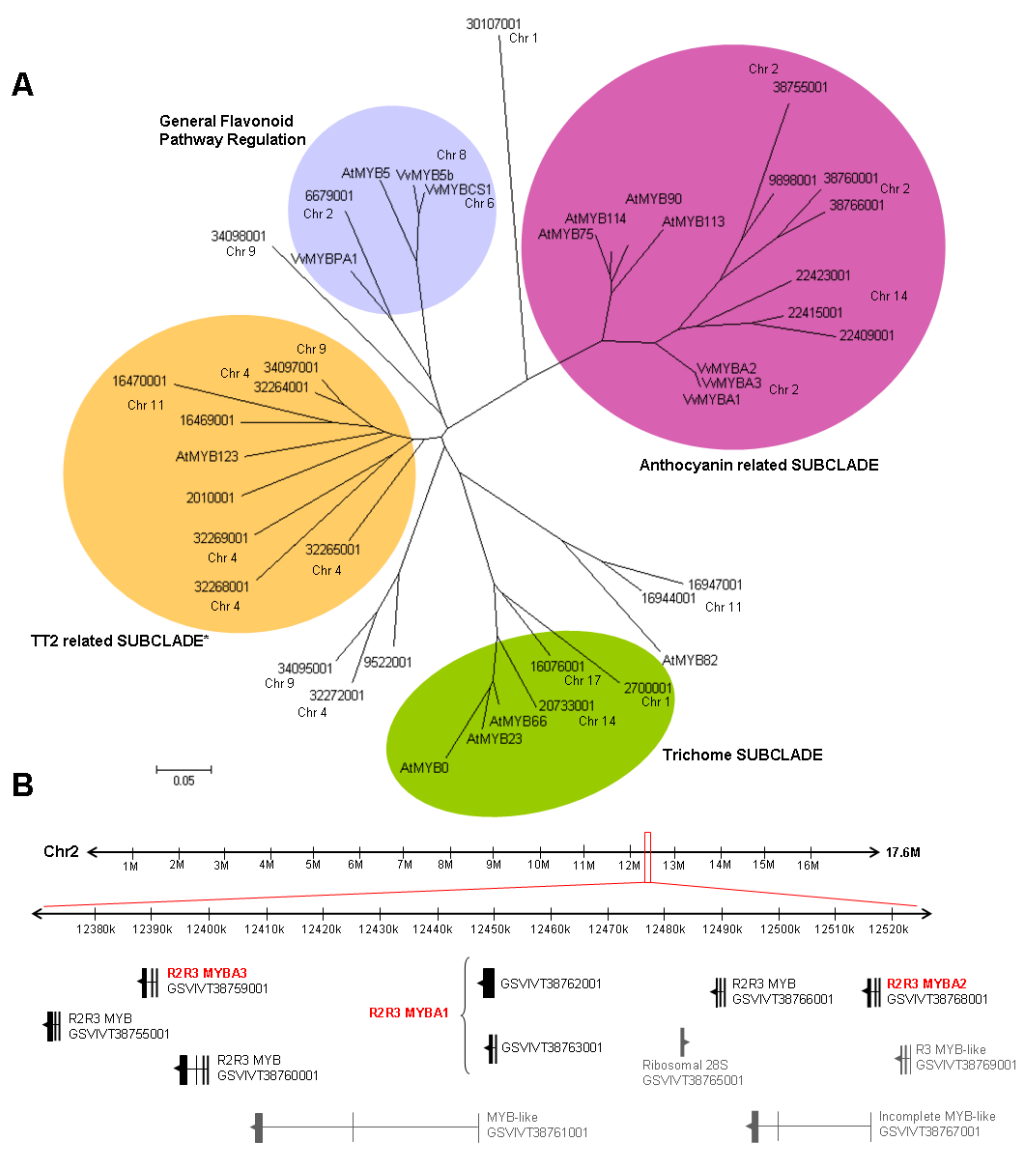
**Phylogenetic tree of the *Vitis*-*Arabidopsis *'epidermal cell fate' clade (A) and anthocyanin related clusters identified in chromosome 2 (B)**. R2R3 DNA-binding domain sequences were used to construct a parsimony unrooted tree with Mega4 software using the Neighbour Joining (NJ) tree method. Branch lengths appear in the same units as those of the evolutionary distances used to infer the phylogenetic tree. Gene model IDs refer to the Genoscope annotation (GSVIVT).

The *Vitis*-*Arabidopsis *phylogenetic tree shows that there are nine anthocyanin-related MYB gene models distributed on chromosomes 2 and 14 from grape, the most abundant MYB-containing chromosomes (Figure 5A). An additional gene model (GSVIVT 9898001) has yet to be linked to a specific chromosome. Recently, a grape colour locus was described [60], which we now localised on chromosome 2. In the previous study, four *R2R3 MYB *genes were found at this locus, but only two (*VvMYBA1 *and *VvMYBA2) *had a regulatory effect on fruit colour. These proteins differ in the lengths of their C-regions: MYBA2 has a duplicated C-terminal repeat when compared to MYBA1, while MYBA3 almost completely lacks this region. These differences could cause novel or cooperative functions, and it has been suggested that VvMYBA3 could control anthocyanin synthesis in grape tissues other than the fruits [60]. In addition to the *VvMYBA1-3 *genes, three other *R2R3 MYB *gene models were detected in the grape colour locus located on chromosome 2 (Figure 5B, between 12 and 13 Mb. Note that MYBA1 was annotated as two contiguous gene models: GSVIVT38763001 and 38762001).

Chromosome 14 contains three anthocyanin related MYBs, some of them being predicted as possible orthologues of the *Arabidopsis *anthocyanin-related AtMYB75 and AtMYB90 proteins (Figure 5A). Further experiments are required to determine whether these genes are functional, in order to verify if this new locus influences the colouring of fruits or other plant organs.

In concordance with the *Arabidopsis *chromosomal duplications classified by Bowers et al [61], *MYB *gene evolution is though to involve segmental duplications [5]. The *Arabidopsis *'anthocyanin-related' subclade, which includes AtMYB75, 90 [62], 113 and 114 [63] (all positioned on chromosome 1), was described as a cluster which may have arise by tandem duplications (see Supplementary material in [5]). The anthocyanin and trichome-related subclades within the *Arabidopsis *'epidermal cell fate' clade may have experienced a major rapid expansion after diverging from monocots but before separating from other dicots [5]. Supporting this hypothesis is the finding that these two clusters are absent in the rice subfamily (Additional file 5). Considering the homology and orientation of the nine anthocyanin related MYBs found in the two grape loci, it is possible that some of the original MYBs could have undergone new multiple tandem duplication events somewhere before or after the separation of the *Arabidopsis*-*Vitis *lineages. However, if this event occurred before lineage separation, this second expansion has clearly been lost during the subsequent evolution of the *Arabidopsis *genome.

Seed pigmentation is defined by the accumulation and oxidation of flavan-3-ols, which are transported in the same way as anthocyanins, but are additionally polymerised into proanthocyanidins (PA) and condensed tannins inside vacuoles (reviewed in [64]). In wines, these molecules define astringency and bitterness. AtMYB123 (also known as TT2) controls the final steps of PA synthesis [7]. Our analysis suggests that grapevine possesses several putative *TT2 *orthologues (Figure 5A), many of which are more closely related to *TT2 *than the previously characterised *MYB5a, MYB5b *and *MYBPA1*. These genes control PA synthesis and also regulate other biosynthetic genes of the flavonoid pathway [24-26]. Chromosomes 4, 9 and 11 possess four, one and two grape *TT2 *homologue genes, respectively. MYBPA1/GSVIVT00006679001 and MYB5a/b are shown as dichotomous pairs. Taken together, MYBs related to PA synthesis also appear expanded. The fact that in *Lotus japonicus *the TT2 lineage compromises a three-member subclade [65], addresses the question whether this expansion is exclusive to grape. When including the rice MYB subfamily into this analysis, only two models appeared as putative TT2 orthologues (Additional file 5), indicating that grape has substantially more TT2 loci than other species characterised to date.

The observed new expansion events are specific to the anthocyanin related subclade and the *TT2 *related lineage in grapevine, but not to the trichome subclade. Both the grapevine adaptive responses (because of the adaptative functions of flavonoids) and human breeding and domestication practices could have helped in the selection of grape genotypes with expanded *MYB *genes involved in colour and astringency.

### Selection of grape gene models for expression analysis and modelling of their DNA binding domains

In order to test the annotation procedure and validate gene models, six annotated genes were randomly selected, isolated and their expression patterns were screened in different grapevine organs. VvMYB4 (Genbank accession EF113078), a AtMYB4 repressor orthologue recently characterised in our group was also included. Complete coding regions of VvMYB12-like, 14-like, 24-like, 30-like, 60-like and a C2motif containing repressor-like gene were isolated from different organs of cv. Cabernet-Sauvignon by RT-PCR (Genbank accessions in Additional file 6). The cDNA sequences were 95–100% similar to their respective gene models (annotated from cv. Pinot noir).

**Figure 6:**
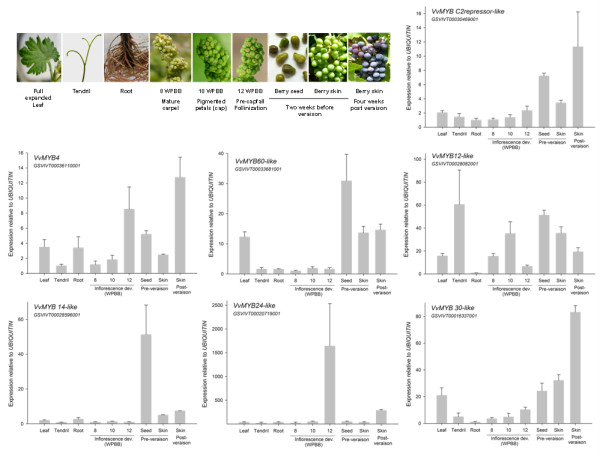
**MYB gene expression profiles in different grape organs and during different stages of inflorescence and berry skin development**. Veraison corresponds to the onset of ripening, when clusters are 30–50% coloured and sugar concentration reaches 5° Brix (5% w/w soluble solids). Standard deviations (SD) are the result of three independent replicates. Weeks post-budbreak (WPBB) is used to follow flower development [74].

Although some *Arabidopsis *MYBs have yet to be functionally characterised, global gene expression data throughout plant development is available on the *Arabidopsis *Electronic Fluorescent Pictograph (eFP) Browser [66,67]. All selected grape *MYB *genes are highly expressed in reproductive organs and revealed organ-expression profiles remarkably similar to their putative *Arabidopsis *orthologues (Figure 6 and Additional file 7). In addition, *VvMYB24 *and *14-like *genes are expressed in a tissue-specific manner in ripped berries/inflorescences and seeds, respectively, in a similar way as in *Arabidopsis*. Subsequent experiments are needed to examine the precise function of these putative orthologies.

A comparative modelling approach (SWISSMODEL) using crystallised mammalian c-MYB (1GV2.PDB) as the structural template was conducted in order to study DNA binding domains from the previously selected MYBs. The *in silico *3D model of these R2R3 domains suggest significant structural homology with rat R1R2R3 c-MYB (Additional file 8). Structural studies of the mammalian c-MYB factor show that its DNA binding domain requires several trypthophan residues which participate in the hydrophobic grouping within the DNA-protein interaction interface [68]. These residues are maintained in the grape homologues (as displayed in asterisks in Additional file 8A).

Some subtle differences between the animal and plant MYBs are also present. Animal MYB domains have a single cysteine residue that needs to be reduced for DNA binding and transcriptional activity [69], contrary to most plant R2R3 MYB domains (including grapes) which contain two cysteines (Additional file 8A,C). Under non-reducing conditions, these cysteines form a disulfide bond preventing DNA binding [69]. The second cysteine residue, conserved in both animals and plants, is essential for regulatory activity in animals. Comparatively, the first cysteine, present only in plants, is crucial for MYB function, demonstrating structural and functional differences between plants and animals that could influence the dynamics of the protein-DNA interaction. The residues surrounding these cysteines are highly similar in the grape MYB domains analysed, further suggesting the importance of their conservation.

Turns between the α2 and α3 helices in each repeat (Additional file 8B, middle and right panels) are essential for conferring globular-like structures (Additional file 8C). Grape R2R3 MYB domains are mostly conserved in the DNA-interacting R2α3, R3α1 and R3α2 helices and turns, as well as in their charge. The most distinct domains in terms of general architecture and positive net charge (yellow regions in each of the structures) were those from VvMYB4 and VvMYBC2 repressor-like proteins, possibly due to their mechanism of action; it has been postulated that MYB repressors affect other MYBs in their binding by either protein-protein interactions or as dominant negative factors [70].

## Conclusion

The sequencing of the grape genome constituted a powerful tool for gene search and evolution studies. Many protein families have been studied in terms of genome conservation across species, in order to establish a genome evolution scenario regarding duplication events and functional diversification. As an example, exon-intron boundaries, expression patterns and regulatory elements in plant D-type cyclin promoters were found to be strongly conserved between *Arabidopsis *and poplar genomes [71]. The identification of grape *MYB *genes and their comparative analysis with the *Arabidopsis *genome suggests strong conservation but also expansions of particular functional clades. Despite recent events of diversification, the exon/intron organisation of *MYB *genes is highly conserved, suggesting restrictions in R2R3 domain variations while allowing C-terminal modifications for gaining new or cooperative functions.

The identification of *MYB *genes in grapevine opens the possibility of modulating *MYB *gene expression in order to control specific aspects of grape physiology and development. In particular, genes involved in flower and seed development or in flavonoid synthesis might be interesting targets for functional characterisation in light of expression similarities in *Arabidopsis *and their possible duplication histories.

## Methods

### Search for MYB homologues in the Grape Genome and Isolation of Candidate genes

The consensus MYB R2R3 DNA binding domain sequence, obtained from the alignment of previously-isolated and characterised genes from *Vitis vinifera *and *Arabidopsis *(Additional file 1), was used in a BLATsearch to identify homologous gene models in the Grape Genome Browser, designed by Genoscope (the French National Sequencing Center [29]). The results obtained were compared to tentative consensus (TC) sequences available at the Grape TIGR Gene Index EST-database.

### Phylogeny reconstruction and bootstrap analysis

To place the obtained sequences in a phylogenetic context, a phylogeny reconstruction was performed with the single DNA-binding domain and full predicted amino acid sequences of grape *MYB *genes and then integrated in the *Arabidopsis *phylogenetic tree. Alignments were performed using the BLOSUM matrix (Gap opening and extension penalties of 25 and 1, respectively) using the ClustalW algorithm-based AlignX module from Mega4 software [72]. The phylogenetic tree was constructed using the Neighbour Joining Tree Method in Mega4 and confirmed with Mega3. Tree nodes were evaluated by bootstrap analysis for 1000 or 2000 replicates. All positions containing gaps and missing data were eliminated from the dataset (Complete deletion option in Mega4 software). Evolutionary distances were computed using the Poisson correction method and are represented as amino acid substitutions per site. Sequences of all 126 *Arabidopsis *R2R3 MYB proteins were downloaded from the TAIR *Arabidopsis *genome annotation version 7.0, released on April 2007.

### Plant materials, RNA isolation and cDNA synthesis

Seven *MYB *candidate genes were isolated from different cDNAs using specific primers (Additional file 7). Different vegetative and reproductive tissues were collected from grapevine plants (*Vitis vinifera *L. cv. Cabernet-Sauvignon) growing in commercial fields in central Chile. Total RNA was isolated according to the procedure of Reid et al [73], using a CTAB-spermidine extraction buffer. For cDNA synthesis, one μg of total RNA was reverse transcribed with random hexamer primers in an 18 μl reaction mixture using the StrataScript^® ^reverse transcriptase (Statagene) according to the manufacturer's instructions. Standard PCR profiles (35 cycles of 30 sec. at 95°C for denaturing, 30 sec. at 57°C for annealing and 1 min at 72°C for extension) were used to amplify *MYB *genes. Amplified fragments were cloned in TOPO-SD cloning vector (Invitrogen, USA), checked for insertion by PCR amplification and sequenced.

### Expression of grape MYB genes in grape organs

Relative transcript quantification of *MYB *genes was achieved by real time RT-PCR, using the Brilliant^® ^SYBR^® ^Green QPCR Master Reagent Kit (Stratagene) and the Mx3000P detection system (Stratagene) as described in the manufacturer's manual. PCR conditions, standard quantification curves for each gene and relative gene expression calculations were conducted according to [74]. Amplification of the *UBIQUITIN1 *gene (99 bp; TC53702, TIGR database, VvGi5) was used for normalisation. The ratio between the gene of interest (*GOI*) and *UBIQUITIN *expression was calculated using the equation [1]:

(1) $$\frac{{\left(1+{\text{E}}_{\text{GOI}}\right)}^{-\Delta \text{Ct}}}{{\left(1+{\text{E}}_{\text{Ubiquitin}}\right)}^{-\Delta \text{Ct}}}=\frac{{\left(1+{\text{E}}_{\text{GOI}}\right)}^{-(\text{Ct GOI}-\text{Ct GOI calibrated})}}{{\left(1+{\text{E}}_{\text{Ubiquitin}}\right)}^{-(\text{Ct Ubi}-\text{Ct Ubi calibrated})}}$$

where E corresponds to each primer amplification efficiency value. Gene expression levels were normalised to that of the organ sample with the lowest expression in order to obtain a calibrated ΔCt for each gene.

### Comparative modelling of grape R2R3 MYB DNA binding domain structures

Using the *Ratus sp*. c-MYB crystal structure (1GV2.pdb) as a template, grape MYB structures were produced by SWISSMODEL [75] and viewed using 3D Molecule Viewer. Charge distributions were analysed in each structure generated.

## Authors' contributions

JTM carried out the genome search, sequence, phylogenetic, expression and structure analyses and drafted the manuscript. FA contributed to phylogenetic analysis, the comparative modelling and together with JTM contributed to the conception of the study. PA–J was involved in revising the manuscript critically for important intellectual content and gave final approval of the version to be published. All authors read and approved the final manuscript.

## Supplementary Material

Additional file 1 **Multiple alignment of 20 representative R2R3 MYB domains from *Arabidopsis *and six MYB domains from characterised grape *MYB *genes**. Identical amino acid residues are shaded in yellow and the blue and white boxes indicate the extent of the R2 and R3 repeats. The consensus sequence shown under the alignment was used to search for *MYB *homologues in the Grape Genome. Click here for file

Additional file 2 List of *R2R3 MYB *gene models in the Grape Genome with their predicted orthologues in sequenced plant species and exon lengths. Click here for file

Additional file 3 **List of *R2R3 MYB *genes in the *Arabidopsis *Genome, including their chromosome positions and protein and exon lengths**. *AtMYB88 *and *AtMYB124 *are highlighted since they possess 10 and 11 exons, respectively and only the first five exons appear in the table. Click here for file

Additional file 4 **Complete phylogenetic tree of *Vitis *and *Arabidopsis *MYB proteins, using the DNA-binding domain (A) or full protein sequences (B)**. The Neighbour Joining (NJ) tree method was used. Numbers above nodes represent bootstrap values for 2000 replicates. Asterisks indicate *Vitis*-specific gene pairs and red and blue letters next to gene model identifiers refer to Genoscope orthologue and Blastp homologue predictions, respectively. Click here for file

Additional file 5 **Phylogenetic tree of the Vitis , Arabidopsis and Rice R2R3 MYB Subfamily**. The R2R3 DNA-binding domain sequences were used for the construction of a parsimony phylogeny tree with Mega4 software using the Neighbour Joining (NJ) tree method. Numbers above nodes represent bootstrap values for 2000 replicates. Click here for file

Additional file 6 Selected candidate grape *MYB *genes and PCR primers used for isolation and real-time Q-PCR. Click here for file

Additional file 7 **Affymetrix (ATH1 Gene Chip) MYB expression data during *Arabidopsis *organ development from the putative homologues of the selected grape genes**. Data was collected using the *Arabidopsis *Electronic Fluorescent Pictograph (eFP) Browser. Colours refer to an absolute expression unit, calculated independently for each gene and normalised by the RMA or GCOS method (eFP Browser by Vinegar, drawn by J.Alls and N. Provart. Data from Gene Expression Map of *Arabidopsis *Development: [66,67], and the Nambara lab for the imbibed and dry seed stages). Click here for file

Additional file 8 **Three dimensional modelling of grape MYB R2R3 domains**. A) *Vitis *and *Arabidopsis *genes were aligned with the *Ratus *c-MYB R2R3 domain using the ClustalW module (VECTOR NTI, Invitrogen). Identical amino acid residues are shaded in yellow, and bars indicate the positions of the three α-helices from each repeat. The six asterisks indicate the constantly spaced tryptophan residues. The arrowhead indicates residues previously described as necessary or dependent for MYB-bHLH interaction. B) Ribbon diagram of the *Ratus *c-MYB R2R3 domain. The DNA-interaction interface is shown (left panel), in which basic residues (coloured in yellow) are exposed outside the R2-α3, R3-α2 and R3-α3 helices (middle panel). Left and middle panels show R2R3 domain in the backwise orientation, as indicated by a black arrow showing the initial R2 residue. C) Charge distributional ribbon diagrams of the R2R3 domain from grape MYBCS-1, MYBC2 repressor like and MYB14 like proteins, obtained by comparative modelling. Residues in volume correspond to cys (green), arg (orange), leu (brown), lys (yellow), gly (blue), thr (cyan), glu (magenta) and ala (grey). Non-volume residues in yellow correspond to positively charged amino acids (arg, lys, his). Click here for file
