# Supplementary figures and images for: Analysis of the grape MYB R2R3 subfamily reveals expanded wine quality-related clades and conserved gene structure organization across Vitis and Arabidopsis genomes
Source: BMC Plant Biol. 2008 Jul 22;8:83. doi: 10.1186/1471-2229-8-83 (PMC2507771; doi:10.1186/1471-2229-8-83)

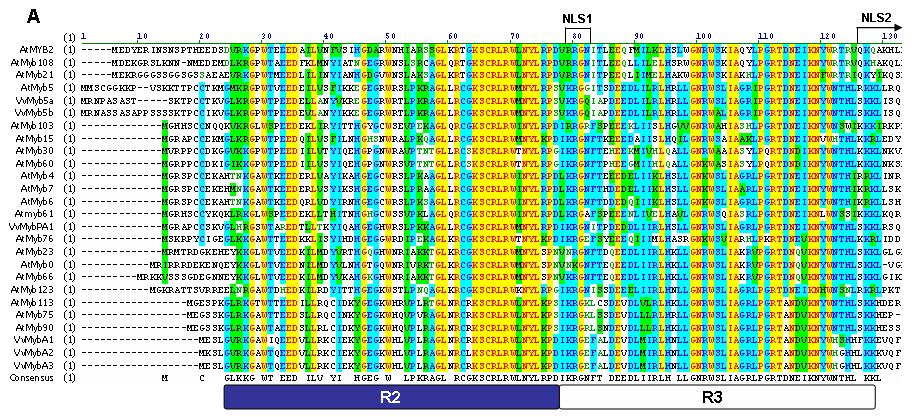

Supplement: Additional file 1 — Multiple alignment of 20 representative R2R3 MYB domains from Arabidopsis and six MYB domains from characterised grape MYB genes. Identical amino acid residues are shaded in yellow and the blue and white boxes indicate the extent of the R2 and R3 repeats. The consensus sequence shown under the alignment was used to search for MYB homologues in the Grape Genome. [file 1471-2229-8-83-S1.tiff]

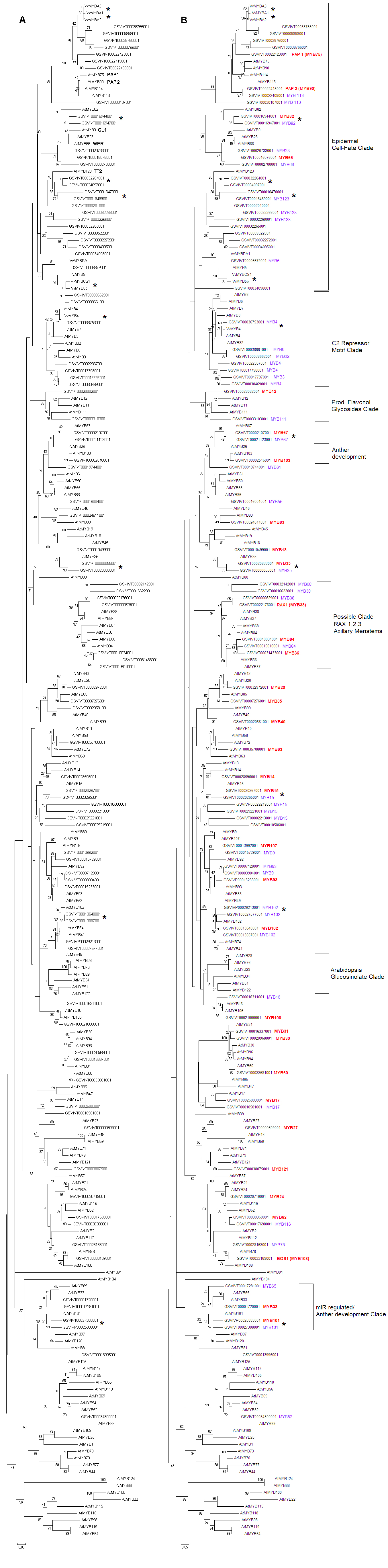

Supplement: Additional file 4 — Complete phylogenetic tree of Vitis and Arabidopsis MYB proteins, using the DNA-binding domain (A) or full protein sequences (B). The Neighbour Joining (NJ) tree method was used. Numbers above nodes represent bootstrap values for 2000 replicates. Asterisks indicate Vitis-specific gene pairs and red and blue letters next to gene model identifiers refer to Genoscope orthologue and Blastp homologue predictions, respectively. [file 1471-2229-8-83-S4.tiff]

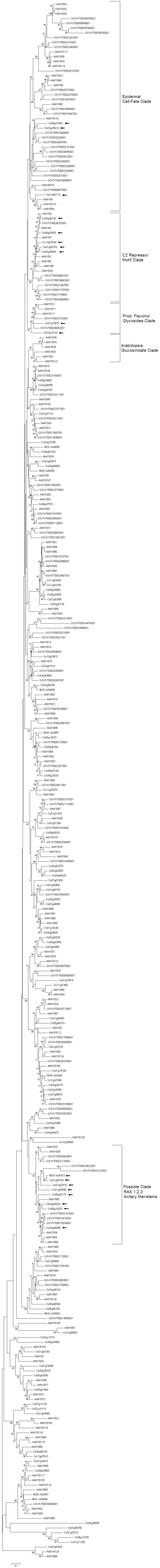

Supplement: Additional file 5 — Phylogenetic tree of the Vitis , Arabidopsis and Rice R2R3 MYB Subfamily. The R2R3 DNA-binding domain sequences were used for the construction of a parsimony phylogeny tree with Mega4 software using the Neighbour Joining (NJ) tree method. Numbers above nodes represent bootstrap values for 2000 replicates. [file 1471-2229-8-83-S5.tiff]

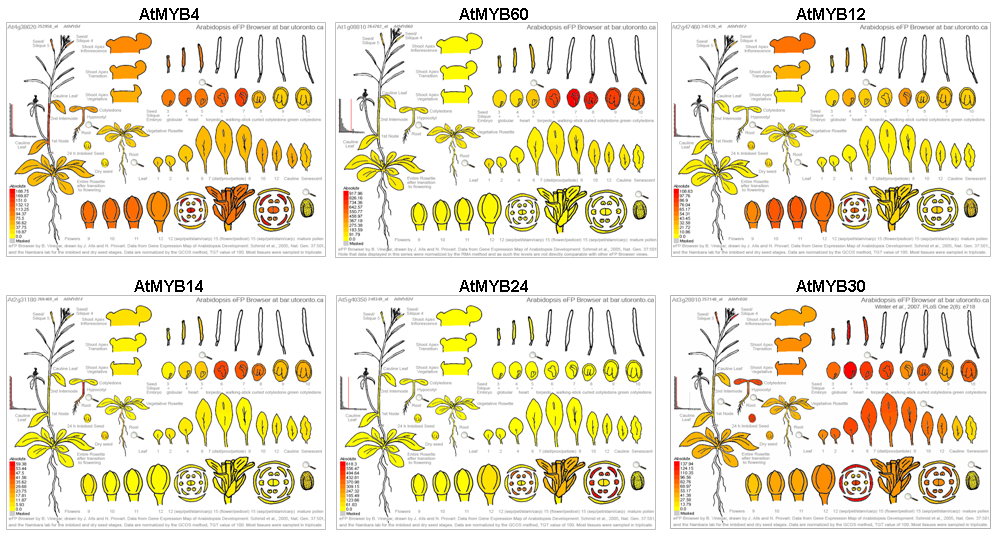

Supplement: Additional file 7 — Affymetrix (ATH1 Gene Chip) MYB expression data during Arabidopsis organ development from the putative homologues of the selected grape genes. Data was collected using the Arabidopsis Electronic Fluorescent Pictograph (eFP) Browser. Colours refer to an absolute expression unit, calculated independently for each gene and normalised by the RMA or GCOS method (eFP Browser by Vinegar, drawn by J.Alls and N. Provart. Data from Gene Expression Map of Arabidopsis Development: [66,67], and the Nambara lab for the imbibed and dry seed stages). [file 1471-2229-8-83-S7.png]

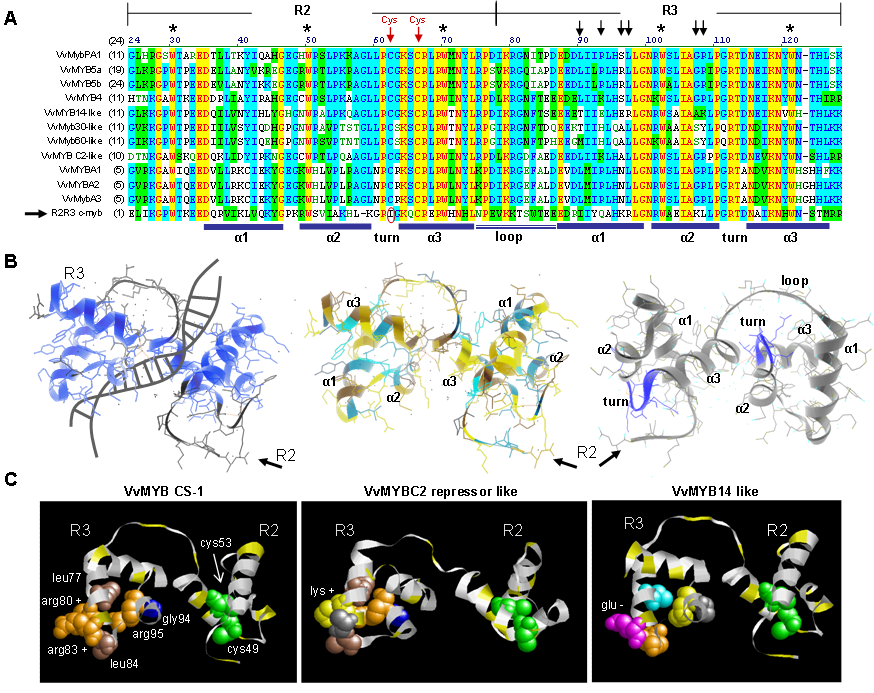

Supplement: Additional file 8 — Three dimensional modelling of grape MYB R2R3 domains. A) Vitis and Arabidopsis genes were aligned with the Ratus c-MYB R2R3 domain using the ClustalW module (VECTOR NTI, Invitrogen). Identical amino acid residues are shaded in yellow, and bars indicate the positions of the three α-helices from each repeat. The six asterisks indicate the constantly spaced tryptophan residues. The arrowhead indicates residues previously described as necessary or dependent for MYB-bHLH interaction. B) Ribbon diagram of the Ratus c-MYB R2R3 domain. The DNA-interaction interface is shown (left panel), in which basic residues (coloured in yellow) are exposed outside the R2-α3, R3-α2 and R3-α3 helices (middle panel). Left and middle panels show R2R3 domain in the backwise orientation, as indicated by a black arrow showing the initial R2 residue. C) Charge distributional ribbon diagrams of the R2R3 domain from grape MYBCS-1, MYBC2 repressor like and MYB14 like proteins, obtained by comparative modelling. Residues in volume correspond to cys (green), arg (orange), leu (brown), lys (yellow), gly (blue), thr (cyan), glu (magenta) and ala (grey). Non-volume residues in yellow correspond to positively charged amino acids (arg, lys, his). [file 1471-2229-8-83-S8.tiff]
