# Supplementary material for: Analysis of the grape MYB R2R3 subfamily reveals expanded wine quality-related clades and conserved gene structure organization across Vitis and Arabidopsis genomes
Source: BMC Plant Biol. 2008 Jul 22;8:83. doi: 10.1186/1471-2229-8-83 (PMC2507771; doi:10.1186/1471-2229-8-83)
Supplement: Additional file 2 — List of R2R3 MYB gene models in the Grape Genome with their predicted orthologues in sequenced plant species and exon lengths. [file 1471-2229-8-83-S2.pdf]

Additional file 2 *. List of R2R3 MYB annotated genes in the Grape Genome with their possible orthologues in sequenced plant species*

BLAT Search using R2R3Myb consensus sequence							Description for each Annotated Identifier							
Annotated Identifier by Genoscope(1)	BLAT Score (2)	Subject region used for LA (3) Start	End	LA % Identity	Chromosome (size)	Str (4)	Position on Chr	Orthologue prediction by Genoscope	Protein Length	Length per exon 1	2	3	4	5
GSVIVT00030469001	53	35	106	87.5%	Chr1 (15630816)	++	4311143.. 4312801	*P trichocarpa* 566512	225	130	130	418		
GSVIVT00030108001	28	35	78	81.9%		++	999398.. 1009443	-	80	57	43	143		
GSVIVT00030360001	33	35	100	75.8%		++	3358494.. 3359580	AtMYB62 (AT1G68320)	288	148	130	589		
GSVIVT00030107001	31	34	78	84.5%		++	996625.. 998672	-	225	145	142	391		
GSVIVT00021123001	25	34	106	67.2%		+-	10921708.. 10922822	*Oryza sativa* 07_03_0483	345	130	908			
GSVIVT00036919001	25	35	77	79.1%	Chr1_random (5496190)	+-	1125809.. 1127755	-	207	133	130	361		
GSVIVT00002700001	23	34	78	75.6%		++	326291.. 327392	-	220	118	130	415		
GSVIVT00006679001	54	35	106	87.5%	Chr2 (17603400)	+-	1800939.. 1801850	*P trichocarpa* 774521	271	133	683			
GSVIVT00015729001	53	2	105	76.0%		+-	7975498.. 7976604	-	335	263	745			
GSVIVT00038769001	35	35	98	78.2%		+-	12521876.. 12522634	-	72	40	134	45		
GSVIVT00038755001	-	-	-	-		+-		-	89	115	134	21		
GSVIVT00038759001 **VvMYBA3 (ABL14067)**	26	34	77	79.6%		+-	12388746.. 12389372	-	266	115	130	556		
GSVIVT00038760001	-	-	-	-		+-		-	205	115	134	99	270	

GSVIVT00038762001 **VvMYBA1 (ABL14063)**	-	-	-	-		+-	12448352.. 12449010	-	250	115	130	508	
GSVIVT00038763001 **VvMYBA1 (ABL14063)**	26	34	77	79.6%		+-	12449064.. 12449458	-	97	115	179		
GSVIVT00038766001	-	-	-	-	Chr2 (17603400)			-	97	115	134	45	
GSVIVT00002546001	23	35	77	76.8%		+-	241759.. 243376	AtMYB103 (AT1G63910)	304	133	130	652	
GSVIVT00007276001	21	78	106	86.3%		+-	4813722.. 4815373	AtMYB85 (AT4G22680)	268	263	544		
GSVIVT00038768001 **VvMYBA2 (ABL14065)**	-	-	-	-		+-	12516756.. 12517438	-	154	115	118	232	
GSVIVT00036110001 **VvMYB4 (EF113078)**	62	4	106	80.6%	Chr3 (10186927)	+-	1569838.. 1571267	AtMYB4 (AT4G38620)	251	263	493		
GSVIVT00036753001	60	4	106	79.7%		+-	15106462.. 15107594	*Oryza sativa* 08_02_1496	242	263	466		
GSVIVT00032265001	53	34	105	87.5%		++	1441131.. 1442325	-	276	133	130	568	
GSVIVT00032266001	53	34	105	87.5%		++	1442841.. 1459186	-	241	20	20	130	556
GSVIVT00032269001	52	34	104	87.4%		++	1469159.. 1470016	-	226	130	130	421	
GSVIVT00032272001	46	35	105	83.1%	Chr4 (19293076)	+-	1483857.. 1484857	*P trichocarpa* 668851	197	136	130	328	
GSVIVT00032264001	32	34	77	86.4%		+-	1420106.. 1421806	*P trichocarpa* 415884	265	133	130	535	
GSVIVT00032268001	28	34	77	81.9%		++	1466509.. 1467957	-	270	130	130	553	
GSVIVT00032142001	21	35	61	88.9%		++	552989.. 553782	-	118	136	221		
GSVIVT00034800001	15	40	96	66.0%		++	18268662.. 18269870	-	302	154	755		

GSVIVT00033189001	29	35	100	72.8%		+-	1570203..<br>1571710	AtMYB108-BOS1<br>(AT3G06490)	306	157	130	634		
GSVIVT00020265001	29	34	78	82.3%		++	7897789..<br>7899269	*Oryza sativa*<br>04_04_0514	267	133	130	541		
GSVIVT00020267001	28	34	77	81.9%	Chr5<br>(23428299)	++	7908863..<br>7910462	AtMYB15<br>(AT3G23250)	253	133	130	499		
GSVIVT00019744001	23	35	79	75.6%		++	2777145..<br>2778204	-	297	133	130	631		
GSVIVT00033103001	19	35	77	72.1%		+-	803644..<br>805722	-	383	133	130	889		
GSVIVT00024797001<br>**VvMYB5b (AAX51291)**	41	1	77	76.7%		++	728440..<br>729634	*Oryza sativa*<br>01_06_0367	312	302	637			
GSVIVT00017281001	35	2	100	68.9%	Chr6<br>(24148918)	+-	14294469..<br>14299041	-	460	336	1047			
GSVIVT00024611001	30	2	78	76.8%		++	2562202..<br>2564124	AtMYB83<br>(AT3G08500)	325	296	682			
GSVIVT00022176001	18	35	64	80.0%		+-	22102247..<br>22117492	AtMYB38<br>(AT2G36890)	299	136	130	500	134	
**GSVIVT00028596001**	28	35	78	81.9%		++	6618108..<br>6621275	AtMYB14<br>(AT2G31180)	269	133	130	547		
GSVIVT00010586001	27	35	104	70.0%	Chr7<br>(15233747)	+-	10332185..<br>10333643	-	102	136	134	39		
**GSVIVT00028082001**	24	35	78	77.3%		+-	4096409..<br>4098680	AtMYB12<br>(AT2G47460)	367	133	130	841		
GSVIVT00028163001	16	35	78	68.2%		+-	4751031..<br>4752072	-	285	151	130	577		
GSVIVT00025459001<br>**VvMYBCs1(AAS68190)**	41	1	77	80.6%		+-	19879628..<br>19880963	AtMYB5<br>(AT3G13540)	320	287	676			
GSVIVT00000609001	33	34	104	82.4%	Chr8<br>(21557227)	+-	17790635..<br>17791607	AtMYB27<br>(AT3G53200)	123	115	130	127		
**GSVIVT00033681001**	27	35	77	81.4%		++	1265357..<br>1266809	AtMYB60<br>(AT1G08810)	279	133	130	90	235	252

GSVIVT00000629001	18	35	64	80.0%		++	17944682..<br>17946528	*P trichocarpa*<br>273997	342	136	130	763	
GSVIVT00034097001	55	34	105	88.9%		+-	1171381...<br>1172557	-	301	133	130	643	
GSVIVT00034098001	47	35	106	83.4%	Chr9<br>(16532244)	+-	1182783..<br>1184304	-	263	133	130	529	
GSVIVT00034095001	49	34	105	84.8%		++	1158346..<br>1159150	*P trichocarpa*<br>582403	203	154	130	328	
GSVIVT00016466001	52	35	105	87.4%		+-	1020903..<br>1021733	-	230	50	643		
GSVIVT00016944001	43	35	104	81.5%		++	5080530..<br>5081411	AtMYB82<br>(AT5G52600)	223	136	130	406	
GSVIVT00016470001	30	34	77	84.1%		+-	1041702..<br>1043283	-	133	133	269		
GSVIVT00016622001	22	35	64	86.7%	Chr11<br>(13936303)	+-	2170576..<br>2171358	-	104	136	134	45	
GSVIVT00016947001	21	78	104	88.9%		++	5098789..<br>5099813	-	269	274	130	406	
GSVIVT00016942001	19	41	77	75.7%		++	5066573..<br>5067111	-	142	23	406		
GSVIVT00031433001	18	35	60	84.7%		++	13389175..<br>13390645	AtMYB36<br>(AT5G57620)	366	136	123	13	829
GSVIVT00016469001	52	35	105	87.4%		+-	1032035..<br>1033915	-	220	133	130	400	
GSVIVT00029213001	39	35	106	77.8%		++	10101435..<br>10102634	-	334	133	130	742	
GSVIVT00029221001	39	35	106	77.8%	Chr12<br>(18540817)	+-	10183659..<br>10184852	*P trichocarpa*<br>239794	257	133	130	511	
GSVIVT00029219001	36	35	104	84.7%		+-	10171223..<br>10172581	-	252	133	130	496	
GSVIVT00025883001	34	1	100	68.1%		++	7290073..<br>7291534	AtMYB101<br>(AT2G32460)	426	336	945		

GSVIVT00028808001	25	34	78	77.8%	Chr13 (15191948)	+-	14610873.. 14612392	-	289	188	682			
GSVIVT00001720001	15	86	100	100.0%		+-	10141782.. 10147862	AtMYB33 (AT5G06100)	551	363	1009	123	161	
GSVIVT00020733001	46	34	106	82.2%		+-	16909499.. 16910521	-	217	118	130	406		
GSVIVT00020833001	33	35	106	73.7%		++	17588097.. 17589575	*At* MYB35 (AT3G28470)	320	133	130	544	156	
GSVIVT00020968001	26	35	78	79.6%		+-	18699080.. 18700450	AtMYB30 (AT3G28910)	327	133	130	721		
GSVIVT00021000001	25	35	77	79.1%	Chr14 (19480434)	++	18963442.. 18965217	AtMYB106 (AT3G01140)	330	133	130	352	42	336
GSVIVT00032972001	21	34	58	92.0%		+-	8392290.. 8401583	AtMYB20 (AT1G66230)	289	133	130	607		
GSVIVT00020581001	20	35	82	70.9%		++	15556689.. 15558153	AtMYB40 (AT5G14340)	268	133	130	544		
**GSVIVT00020719001**	18	35	78	70.5%		++	16764197.. 16768191	AtMYB24 (AT5G40350)	189	136	130	304		
GSVIVT00038075001	18	77	104	82.2%		+-	12582574.. 12583545	AtMYB121 (AT3G30210)	247	142	130	472		
GSVIVT00022367001	52	34	106	86.4%		++	2966946.. 2967891	-	228	130	130	427		
GSVIVT00022409001	44	35	105	81.7%		+-	3849020.. 3850240	-	263	169	148	475		
GSVIVT00022423001	42	35	105	80.3%	Chr14_random (5432426)	+-	4288060.. 4290721	AtMYB75-PAP1 (AT1G56650)	243	118	139	475		
GSVIVT00022415001	30	35	78	84.1%		+-	4047444.. 4048716	AtMYB90-PAP2 (AT1G66390)	246	118	139	484		
GSVIVT00002213001	25	35	77	79.1%		++	913314.. 914803	-	95	133	134	21		
GSVIVT00026803001	48	1	77	81.9%	Chr15_random (4297576)	++	2162264.. 2165161	AtMYB17 (AT3G61250)	302	133	130	646		

GSVIVT00013992001	37	1	77	74.1%	Chr16 (8158851)	+-	1351513.. 1364135	AtMYB107 (AT3G02940)	330	257	736	
GSVIVT00013995001	8	34	77	59.1%		+-	1411388.. 1435018	-	169	248	211	51
GSVIVT00008781001	19	78	106	82.8%	Chr16_random (4524411)	++	3555527.. 3557038	-	261	154	73	559
GSVIVT00017798001	56	34	106	89.1%	Chr17 (13059092)	+-	2459123.. 2461057	-	175	130	130	268
GSVIVT00017797001	55	35	106	88.9%		+-	2464208.. 2465491	-	229	178	130	382
GSVIVT00017698001	31	35	100	74.3%		+-	3444848.. 3446270	-	328	151	130	706
**GSVIVT00016337001**	26	35	78	79.6%		++	6869110.. 6870718	AtMYB31 (AT1G74650)	307	133	130	661
GSVIVT00016311001	25	35	77	79.1%		++	7086341.. 7087632	-	330	133	130	730
GSVIVT00016004001	22	35	78	75.0%		++	10759745.. 10761118	*P trichocarpa* 423254	367	133	130	841
GSVIVT00016076001	22	35	78	75.0%		+-	9813627.. 9814524	AtMYB66-WER (AT5G1475)	216	118	130	403
GSVIVT00000055001	20	35	78	72.8%		+-	5917584.. 5919123	*Oryza sativa* 03_02_0675	337	143	120	751
GSVIVT00015010001	34	35	106	80.4%	Chr18 (19691255)	++	6829941.. 6831217	*Oryza sativa* 03_06_0112	340	136	130	757
GSVIVT00015233001	20	34	77	72.8%		++	4371454.. 4372597	AtMYB93 (AT1G34670)	324	133	130	712
GSVIVT00038661001	50	35	101	88.1%	Chr18_random (5949186)	+-	3692097.. 3693231	-	237	133	130	451
GSVIVT00038662001	47	35	106	83.4%		+-	3705618.. 3706531	-	238	133	130	454
GSVIVT00027308001	31	2	100	66.7%	Chr19	+-	6638859.. 6640552	-	381	327	819	

GSVIVT00027577001	21	35	77	74.5%	(14071813)	++	3934378.. 3935733	*P trichocarpa* 811245	341	133	130	272	491
GSVIVT00002107001	17	34	78	68.9%	Chr19_random (1912523)	++	1371840.. 1373040	AtMYB67 (AT3G12720)	337	130	130	754	
GSVIVT00008644001 **VvMYBPA1(CAJ90831)**	62	35	106	93.1%		+-	51310430.. 51311315	-	286	133	728		
GSVIVT00009522001	54	34	106	87.7%		++	60623218.. 60624010	-	195	142	130	316	
GSVIVT00002010001	49	34	105	84.8%		++	17409980.. 17410952	-	263	133	130	529	
GSVIVT00008477001	39	35	106	77.8%		++	147357901.. 147359144	-	346	133	130	778	
GSVIVT00013087001	39	35	106	77.8%		+-	84780902.. 84782192	*Oryza sativa* 07_03_0877	362	133	130	826	
GSVIVT00013648001	39	35	106	77.8%		+-	88327164.. 88328454	A*t* MYB102 (AT4G21440)	362	133	130	826	
GSVIVT00007128001	37	35	106	76.4%	Chr Un_random (154883714)	+-	45190481.. 45191693	-	330	133	130	730	
GSVIVT00010501001	28	35	78	81.9%		++	64517813.. 64519819	-	296	133	130	628	
GSVIVT00009898001	27	34	78	80.0%		+-	135757338.. 135757972	-	89	115	134	21	
GSVIVT00035708001	25	35	77	79.1%		+-	132844356.. 132846672	AtMYB63 (AT1G79180)	327	139	130	715	
GSVIVT00003904001	19	35	77	72.1%		+-	89062565.. 89063760	*Oryza sativa* 06_01_0828	330	133	130	730	
GSVIVT00010034001	18	35	60	84.7%		+-	149868463.. 149869714	AtMYB84 (AT3G49690)	339	136	130	754	
GSVIVT00010499001	14	78	105	75.0%		++	64506147.. 64507827	AtMYB18 (AT4G25560)	298	142	130	625	
								Average Prot Length	264.6				

Random chromosomes contains sequences that have been assigned to a particular grape chromosome but their position could not be determined in relation to other sequences. ChrUn_random contains ultracontigs that could not be placed on a certain chromosome. These were joined together into

(1) Gene prediction and data integration was performed by Genoscope with GAZE computational framework (Howe *et al.* , 2002).
(2) BLAT finds sequences of 20 amino acids or more with 80% or higher similarity in the genome, by keeping an index of the entire genome in memory.
(3) LA: Local alignment between query (R2R3Myb DNA-binding domain consensus sequence) and subjects.
(4) Str: The first strand corresponds to the grape genomic sequence and the second corresponds to the query sequence.
