# Supplementary material for: Analysis of the grape MYB R2R3 subfamily reveals expanded wine quality-related clades and conserved gene structure organization across Vitis and Arabidopsis genomes
Source: BMC Plant Biol. 2008 Jul 22;8:83. doi: 10.1186/1471-2229-8-83 (PMC2507771; doi:10.1186/1471-2229-8-83)
Supplement: Additional file 3 — List of R2R3 MYB genes in the Arabidopsis Genome, including their chromosome positions and protein and exon lengths. AtMYB88 and AtMYB124 are highlighted since they possess 10 and 11 exons, respectively and only the first five exons appear in the table. [file 1471-2229-8-83-S3.pdf]

MYB	Length per exon 1	2	3	4	5	ORF bp	Protein Length	**TAIR ID**	Function REF*.
0	139	130	418	-	-	687	228	**AT3G27920.**	Openhaimer et al., 1991
1	334	848	-	-	-	1182	393	**AT3G09230**	-
2	157	130	535	-	-	822	273	**AT2G47190.**	Abe et al., 2003
3	133	130	511	-	-	774	257	**AT1G22640.**	Curated by TAIR
4	263	586	-	-	-	849	282	**AT4G38620.**	Jin et al., 2000
5	296	454	-	-	-	750	249	**AT3G13540.**	Li et al., 1996
6	263	448	-	-	-	711	236	**AT4G09460.**	Li and Parish, 1995
7	263	547	-	-	-	810	269	**AT2G16720.**	Li and Parish, 1995
8	133	130	376	-	-	639	212	**AT1G35515.**	Zhu et al., 2005
9	263	711	37	-	-	1011	336	**AT5G16770.**	-
10	139	130	451	-	-	720	239	**AT3G12820.**	-
11	133	130	769	-	-	1032	343	**AT3G62610.**	Stracke et al., 2007
12	133	130	853	-	-	1116	371	**AT2G47460.**	Mehrtens et al., 2005
13	133	130	478	-	-	741	246	**AT1G06180.**	Kirik et al., 1998
14	133	130	487	-	-	750	249	**AT2G31180.**	-
15	133	130	595	-	-	858	285	**AT3G23250.**	Agarwal et al., 2006
16	133	130	350	368	-	981	326	**AT5G15310.**	Baumann et al., 2007
17	133	130	610	-	-	873	290	**AT3G61250.**	-
18	127	130	595	-	-	852	283	**AT4G25560**	Ballesteros et al., 2001
19	133	130	544	-	-	807	268	**AT5G52260**	-
20	263	586		-	-	849	282	**AT1G66230.**	-
21	157	130	394	-	-	681	226	**AT3G27810.**	Mandaokar et al., 2006
22	188	124	459	-	-	771	256	**AT5G40430**	-
23	133	130	397	-	-	660	219	**AT5G40330.**	Kirik et al., 2001
24	148	130	367	-	-	645	214	**AT5G40350.**	Yang et al., 2007b
25	319	785		-	-	1104	367	**AT2G39880**	-
26	160	130	814	-	-	1104	367	**AT3G13890.**	Yang et al., 2007a
27	124	130	101	362	-	717	238	**AT3G53200**	-
28	133	130	838	-	-	1101	366	**AT5G61420.**	Gigolashvili et al., 2007b
29	133	130	748	-	-	1011	336	**AT5G07690.**	Gigolashvili et al., 2007c
30	133	130	709	-	-	972	323	**AT3G28910.**	Vailleau et al., 2002
31	133	130	730	-	-	993	330	**AT1G74650.**	-
32	263	562	-	-	-	825	274	**AT4G34990.**	Preston et al., 2004
33	345	967	251	-	-	1563	520	**AT5G06100.**	Reyes and Chua, 2007
34	133	130	625	-	-	888	295	**AT5G60890.**	Celenza et al., 2005

35	133	130	691	-	-	954	317	**AT3G28470.**	-
36	136	130	736	-	-	1002	333	**AT5G57620.**	-
37	136	130	724	-	-	990	329	**AT5G23000.**	Keller et al., 2006
38	136	130	631	-	-	897	298	**AT2G36890.**	Müller et al., 2006
39	266	780	37	-	-	1083	360	**AT4G17785.**	-
40	133	130	529	-	-	792	263	**AT5G14340.**	-
41	133	130	586	-	-	849	282	**AT4G28110.**	Cominelli et al., 2007
42	507	-	-	-	-	507	168	**AT4G12350**	-
43	133	130	721	-	-	984	327	**AT5G16600.**	-
44	918	-	-	-	-	918	305	**AT5G67300**	Jung et al., 2007
45	151	130	505	-	-	786	261	**AT3G48920**	-
46	281	562	-	-	-	843	280	**AT5G12870.**	-
47	133	130	541	-	-	804	267	**AT1G18710.**	-
48	118	130	523	-	-	771	256	**AT3G46130**	Li et al., 2006
49	133	130	697	-	-	960	319	**AT5G54230.**	-
50	133	130	682	-	-	945	314	**AT1G57560.**	-
51	136	130	793	-	-	1059	352	**AT1G18570.**	Gigolashvili et al., 2007a
52	118	426	206	-	-	750	249	**AT1G17950**	-
53	133	130	670	-	-	933	310	**AT5G65230.**	-
54	121	432	179	-	-	732	243	**AT1G73410**	-
55	169	130	748	-	-	1047	348	**AT4G01680.**	-
56	382	426	164	-	-	972	323	**AT5G17800**	Curated by TAIR
57	172	130	319	-	-	621	206	**AT3G01530.**	-
58	139	130	556	-	-	825	274	**AT1G16490.**	-
59	121	130	457	-	-	708	235	**AT5G59780**	Li et al., 2006
60	133	130	580	-	-	843	280	**AT1G08810.**	Cominelli et al., 2005
61	133	130	838	-	-	1101	366	**AT1G09540.**	Liang et al., 2005
62	154	130	577	-	-	861	286	**AT1G68320.**	-
63	139	130	616	-	-	885	294	**AT1G79180.**	-
64	341	127	804	-	-	1272	423	**AT5G11050**	-
65	372	1009	281	-	-	1662	553	**AT3G11440.**	Millar and Gubler, 2005
66	145	130	337	-	-	612	203	**AT5G14750.**	Lee and Schiefelbein., 1999
67	163	761	-	-	-	924	307	**AT3G12720.**	-
68	136	130	859	-	-	1125	374	**AT5G65790.**	-
69	160	593	-	-	-	753	250	**AT4G33450**	-
70	930	-	-	-	-	930	309	**AT2G23290**	-
71	281	529	-	-	-	810	269	**AT3G24310.**	-

72	139	130	622	-	-	891	296	**AT1G56160.**	Van der Ent et al., 2008
73	963	-	-	-	-	963	320	**AT4G37260**	-
74	136	130	709	-	-	975	324	**AT4G05100.**	-
75	121	130	496	-	-	747	248	**AT1G56650.**	Borevitz et al., 2000
76	133	130	754	-	-	1017	338	**AT5G07700.**	Gigolashvili et al., 2007c
77	906	-	-	-	-	906	301	**AT3G50060**	Shin et al., 2007
78	175	130	619	-	-	924	307	**AT5G49620.**	-
79	115	130	541	-	-	786	261	**AT4G13480.**	-
80	133	130	700	-	-	963	320	**AT5G56110.**	-
81	309	975	-	-	-	1284	427	**AT2G26960.**	-
82	133	130	343	-	-	606	201	**AT5G52600.**	-
83	317	715	-	-	-	1032	343	**AT3G08500.**	-
84	136	130	667	-	-	933	310	**AT3G49690.**	Müller et al., 2006
85	263	538	-	-	-	801	266	**AT4G22680.**	-
86	133	130	796	-	-	1059	352	**AT5G26660.**	-
87	136	130	652	-	-	918	305	**AT4G37780.**	-
88	108	47	57	73	56	1455	484	**AT2G02820**	Lai et al., 2005
89	271	302	-	-	-	573	190	**AT5G39700**	-
90	121	130	499	-	-	750	249	**AT1G66390.**	Borevitz et al., 2000
91	1104	-	-	-	-	1104	367	**AT2G37630**	Byrne et al., 2000; Nurmberg et al., 2007
92	133	130	742	-	-	1005	334	**AT5G10280.**	-
93	133	130	835	-	-	1098	365	**AT1G34670.**	-
94	133	130	739	-	-	1002	333	**AT3G47600.**	-
95	133	130	553	-	-	816	271	**AT1G74430.**	-
96	133	130	796	-	-	1059	352	**AT5G62470.**	-
97	306	649	215	-	-	1170	389	**AT4G26930.**	-
98	677	127	480	-	-	1284	427	**AT4G18770**	Kasahara et al., 2005
99	287	451	-	-	-	738	245	**AT5G62320.**	-
100	104	127	474	-	-	705	234	**AT2G25230**	-
101	303	859	311	-	-	1473	490	**AT2G32460.**	Reyes and Chua, 2007
102	133	130	790	-	-	1053	350	**AT4G21440.**	Denekamp & Smeekens, 2003
103	133	130	850	-	-	1113	370	**AT1G63910.**	Higginson et al., 2003
104	297	864	-	-	-	1161	386	**AT2G26950**	-
105	424	369	200	-	-	993	330	**AT1G69560**	-
106	137	125	130	353	422	1167	388	**AT3G01140.**	-
107	263	666	37	-	-	966	321	**AT3G02940.**	-
108	154	130	688	-	-	972	323	**AT3G06490.**	Mengiste et al., 2003

109	337	863	-	-	-	1200	399	**AT3G55730**	-
110	304	288	326	-	-	918	305	**AT3G29020**	-
111	133	130	766	-	-	1029	342	**AT5G49330.**	Stracke et al., 2007
112	193	130	409	-	-	732	243	**AT1G48000.**	-
113	121	130	490	-	-	741	246	**AT1G66370.**	Gonzalez et al., 2007
114	121	130	169	-	-	420	139	**AT1G66380.**	Gonzalez et al., 2007
115	500	127	453	-	-	1080	359	**AT5G40360**	-
116	151	130	571	-	-	852	283	**AT1G25340.**	-
117	397	446	-	-	-	843	280	**AT1G26780**	-
118	144	449	127	594	-	1314	437	**AT3G27785**	-
119	341	127	825	-	-	1293	430	**AT5G58850**	-
120	327	1245	-	-	-	1572	523	**AT5G55020.**	-
121	178	130	523	-	-	831	276	**AT3G30210.**	-
122	133	130	739	-	-	1002	333	**AT1G74080.**	Gigolashvili et al., 2007a
123	139	130	508	-	-	777	258	**AT5G35550.**	Nesi et al., 2001
124	93	47	57	73	56	2001	666	**AT1G14350**	Lai et al., 2005
125	188	262	444	-	-	894	297	**AT3G60460**	Rotman et al., 2005
				Average Prot Length			312.0		

*Function REF

Abe H, Urao T, Ito T, Seki M, Shinozaki K, Yamaguchi-Shinozaki K (2003) Arabidopsis AtMYC2 (bHLH) and AtMYB2 (MYB) Function as Transcriptional Activators in Abscisic Acid Signaling. Plant Cell 15: 63-78

Baumann K, Perez-Rodriguez M, Bradley D, Venail J, Bailey P, Jin H, Koes R, Roberts K, Martin C (2007) Control of cell and petal morphogenesis by R2R3 MYB transcription factors. Development 134(9):1691-701

Byrne ME, Barley R, Curtis M, Arroyo JM, Dunham M, Hudson A, Martienssen RA (2000) Asymmetric leaves1 mediates leaf patterning and stem cell function in Arabidopsis. Nature 408(6815):967-71

Cominelli E, Sala T, Calvi D, Gusmaroli G, Tonelli C (2007) Over-expression of the Arabidopsis AtMYB41 gene alters cell expansion and leaf surface permeability. Plant J. PMID: 17971045

Denekamp M, Smeekens SC. (2003) Integration of wounding and osmotic stress signals determines the expression of the AtMYB102 transcription factor gene. Plant Physiol. 132(3):1415-23

Jung C, Seo JS, Han SW, Koo YJ, Kim CH, Song SI, Nahm BH, Choi YD, Cheong JJ. (2007) Overexpression of AtMYB44 enhances stomatal closure to confer abiotic stress tolerance in transgenic Arabidopsis. Plant Physiol. PMID: 18162593

Kasahara RD, Portereiko MF, Sandaklie-Nikolova L, Rabiger DS, Drews GN (2005) MYB98 is required for pollen tube guidance and synergid cell differentiation in Arabidopsis. Plant Cell. 17(11):2981-92

Keller T, Abbott J, Moritz T, Doerner P (2006) Arabidopsis REGULATOR OF AXILLARY MERISTEMS1 controls a leaf axil stem cell niche and modulates vegetative development. Plant Cell 18(3):598-611

Kirik V, Kölle K, Wohlfarth T, Miséra S, Bäumlein H (1998) Ectopic expression of a novel MYB gene modifies the architecture of the Arabidopsis inflorescence. Plant J. 13(6):729-42

Kirik V, Schnittger A, Radchuk V, Adler K, Hülskamp M, Bäumlein H (2001) Ectopic expression of the Arabidopsis AtMYB23 gene induces differentiation of trichome cells. Dev Biol. 235(2):366-77

Lai LB, Nadeau JA, Lucas J, Lee EK, Nakagawa T, Zhao L, Geisler M, Sack FD (2005) The Arabidopsis R2R3 MYB proteins FOUR LIPS and MYB88 restrict divisions late in the stomatal cell lineage. Plant Cell 17(10):2754-67

Lee MM, Schiefelbein J (1999) WEREWOLF, a MYB-related protein in Arabidopsis, is a position-dependent regulator of epidermal cell patterning. Cell 99(5):473-83

Li SF, Parish RW (1995) Isolation of two novel myb-like genes from Arabidopsis and studies on the DNA-binding properties of their products. Plant J. 8(6):963-72

Li SF, Santini JM, Nicolaou O, Parish RW (1996) A novel myb-related gene from Arabidopsis thaliana. FEBS Lett. 379(2):117-21

Nurmberg PL, Knox KA, Yun BW, Morris PC, Shafiei R, Hudson A, Loake GJ (2007) The developmental selector AS1 is an evolutionarily conserved regulator of the plant immune response. Proc Natl Acad Sci USA 104(47):18795-800

Oppenheimer D, Herman P, Sivakumaran S, Esch J, Marks M (1991) A MYB gene required for leaf trichome differentiation in Arabidopsis is expressed in stipules. Cell 67, 483–493

Rotman N, Durbarry A, Wardle A, Yang WC, Chaboud A, Faure JE, Berger F, Twell D. (2005) A novel class of MYB factors controls sperm-cell formation in plants. Curr Biol. 15(3):244-8

Shin R, Burch AY, Huppert KA, Tiwari SB, Murphy AS, Guilfoyle TJ, Schachtman DP (2007) The Arabidopsis transcription factor MYB77 modulates auxin signal transduction. Plant Cell 8:2440-53

Van der Ent S, Verhagen BW, Van Doorn R, Bakker D, Verlaan MG, Pel MJ, Joosten RG, Proveniers MC, Van Loon LC, Ton J, Pieterse CM (2008) MYB72 Is Required in Early Signaling Steps of Rhizobacteria-Induced Systemic Resistance in Arabidopsis. Plant Physiol. PMID: 18218967
