# Supplementary material for: Analysis of the grape MYB R2R3 subfamily reveals expanded wine quality-related clades and conserved gene structure organization across Vitis and Arabidopsis genomes
Source: BMC Plant Biol. 2008 Jul 22;8:83. doi: 10.1186/1471-2229-8-83 (PMC2507771; doi:10.1186/1471-2229-8-83)
Supplement: Additional file 6 — Selected candidate grape MYB genes and PCR primers used for isolation and real-time Q-PCR. [file 1471-2229-8-83-S6.pdf]

Gene ID (Genebank Accession)	**Primer ID**	**Sequence***
VvMYB12 like	QPCR_MYB fw	5´ CAGTGGCCCTCCTGCTCTT
	QPCR_MYB rev	5´ CCATCACTGTCCTCCCATAACC
VvMYB14 like (EU181424)	TOPO_MYB fw	5´ caccATGGGGAGAGCTCCTTGTTGTGAG
	TOPO_MYB rev	5´ TCATATTTCTGATAATTCATGCAA
	QPCR_MYB fw	5´ GGAGAGCCTTGGGTATGGA
	QPCR_MYB rev	5´ CGACCTTAATGCAGGGTGTA
VvMYB24 like (EU181426)	TOPO_MYB fw	5´ caccATGGATAAAAAACCCTGCAATTCTC
	TOPO_MYB rev	5´ TTAATCTCCATTAAGTAGCTGCAT
	QPCR_MYB fw	5´ TCAGACACATGATCAAGCAACT
	QPCR_MYB rev	5´ CGAGATTGGCAGGGTAGGA
VvMYB30 like (EF071984)	TOPO_MYB fw	5´ caccATGGGGAGGCCACCTTGCTG
	TOPO_MYB rev	5´ CTAGAAGAGCTGAGCAGTGT
	QPCR_MYB fw	5´ CTCAAGTCCCTCTCACAATG
	QPCR_MYB rev	5´ TGTCAATTAGGTCTTCTTGTTC
VvMYB60 like (EU816358)	TOPO_MYB fw	5´ caccATGGGAAGGCCTCCTTGCTGTGAT
	TOPO_MYB rev	5´ TCAGAATATTGGAGAGAGTTGATC
	QPCR_MYB fw	5´ TTGAGTACGAAAACCTGAATGAT
	QPCR_MYB rev	5´ GGAGGGTTGTGCTTCTTCTGAT
VvMYBC2 repressor like (EU181425)	TOPO_MYB fw	5´ caccATGAGGAAGCCCTGCTGTGATAAGC
	TOPO_MYB rev	5´ TCATCCAAAGAGGAGCAGCGTGGA
	QPCR_MYB fw	5´ GACCGCAACATCATCTGGTT
	QPCR_MYB rev	5´ GCACGCCGGTTATCATCT
VvMYB4 (EF113078)	QPCR_MYB fw	5´ ACCGGACGTTACAACCATATC
	QPCR_MYB rev	5´ TCCGTAACTGGGTTTTTCTCA

* TOPOSD clonning vector (INVITROGEN) requires the "CACC" sequence in the forward primer
